# Research Infrastructure Contact Zones: a framework and dataset to characterise the activities of major biodiversity informatics initiatives

**DOI:** 10.3897/BDJ.10.e82953

**Published:** 2022-09-16

**Authors:** Vincent Stuart Smith, Lisa French, Sarah Vincent, Matt Woodburn, Wouter Addink, Christos Arvanitidis, Olaf Bánki, Ana Casino, Francois Dusoulier, Falko Glöckler, Donald Hobern, Martin R. Kalfatovic, Dimitrios Koureas, Patricia Mergen, Joe Miller, Leif Schulman, Aino Juslén

**Affiliations:** 1 Natural History Museum, London, United Kingdom Natural History Museum London United Kingdom; 2 Naturalis Biodiversity Center, Leiden, Netherlands Naturalis Biodiversity Center Leiden Netherlands; 3 Distributed System of Scientific Collections - DiSSCo, Leiden, Netherlands Distributed System of Scientific Collections - DiSSCo Leiden Netherlands; 4 LifeWatch ERIC, Seville, Spain LifeWatch ERIC Seville Spain; 5 Institute of Marine Biology, Biotechnology and Aquaculture, Heraklion, Crete, Greece Institute of Marine Biology, Biotechnology and Aquaculture Heraklion, Crete Greece; 6 Catalogue of Life, Amsterdam, Netherlands Catalogue of Life Amsterdam Netherlands; 7 Consortium of European Taxonomic Facilities, Brussels, Belgium Consortium of European Taxonomic Facilities Brussels Belgium; 8 Muséum national d'histoire naturelle, Paris, France Muséum national d'histoire naturelle Paris France; 9 Museum für Naturkunde Berlin, Leibniz Institute for Evolution and Biodiversity Science, Berlin, Germany Museum für Naturkunde Berlin, Leibniz Institute for Evolution and Biodiversity Science Berlin Germany; 10 International Barcode of Life, Guelph, Canada International Barcode of Life Guelph Canada; 11 Smithsonian Institution Libraries and Archives / Biodiversity Heritage Library, Washington, United States of America Smithsonian Institution Libraries and Archives / Biodiversity Heritage Library Washington United States of America; 12 Meise Botanic Garden, Meise, Belgium Meise Botanic Garden Meise Belgium; 13 Royal Museum for Central Africa, Tervuren, Belgium Royal Museum for Central Africa Tervuren Belgium; 14 GBIF, Copenhagen, Denmark GBIF Copenhagen Denmark; 15 Finnish Environment Institute, Helsinki, Finland Finnish Environment Institute Helsinki Finland; 16 University of Helsinki, Helsinki, Finland University of Helsinki Helsinki Finland; 17 Finnish Museum of Natural History, Helsinki, Finland Finnish Museum of Natural History Helsinki Finland

**Keywords:** methods, data visualisation, coordination, alignment, community, biodiversity informatics

## Abstract

**Background:**

The landscape of biodiversity data infrastructures and organisations is complex and fragmented. Many occupy specialised niches representing narrow segments of the multidimensional biodiversity informatics space, while others operate across a broad front, but differ from others by data type(s) handled, their geographic scope and the life cycle phase(s) of the data they support. In an effort to characterise the various dimensions of the biodiversity informatics landscape, we developed a framework and dataset to survey these dimensions for ten organisations (DiSSCo, GBIF, iBOL, Catalogue of Life, iNaturalist, Biodiversity Heritage Library, GeoCASe, LifeWatch, eLTER ELIXIR), relative to both their current activities and long-term strategic ambitions.

**New information:**

The survey assessed the contact between the infrastructure organisations by capturing the breadth of activities for each infrastructure across five categories (data, standards, software, hardware and policy), for nine types of data (specimens, collection descriptions, opportunistic observations, systematic observations, taxonomies, traits, geological data, molecular data and literature) and for seven phases of activity (creation, aggregation, access, annotation, interlinkage, analysis and synthesis). This generated a dataset of 6,300 verified observations, which have been scored and validated by leading members of each infrastructure organisation. The resulting data allow high-level questions about the overall biodiversity informatics landscape to be addressed, including the greatest gaps and contact between organisations.

## Introduction

Biodiversity informatics – the application of informatics techniques and technologies to collate, harmonise, manage, share and use data and information on the world’s biota – has progressed considerably in the last two decades (Gadelha et al. 2020). These developments have made unprecedented volumes of data readily available for the scientific community and other stakeholders. For example, GBIF now offers over 2.2 billion occurrence records (gbif.org; 5 August 2022), providing a comprehensive map of past and present species distributions. iBOL, a global repository of DNA barcodes, now includes 779 thousand barcode index numbers (a proxy for species, boldsystems.org; 5 August 2022). Likewise, the Biodiversity Heritage Library has digitised over 60 million pages of biodiversity literature (biodiversitylibrary.org; 5 August 2022), while citizen science projects like iNaturalist have over 2.3 million observers contributing contemporary data on taxon occurrences (inaturalist.org/observations; 5 August 2022).

Data from these and related infrastructures are critical to addressing many of science and societies greatest challenges, including the interconnected crises of biodiversity loss and climate change. For example, much of the science that underpins policies designed to tackle biodiversity loss comes from data mediated by these infrastructures. Consequently, there is an ever more pressing need to tackle the barriers that hinder the acquisition of more data. As new needs emerge, especially in responding to the growing data needs, ever more coordination is required in the development of new infrastructures. One such gap relates to the provision of data from natural science collections. Their collections provide unique and critical insight into historical distributions of species and are the gateway to a rich wealth of additional information associated with these specimens. At present, very little (perhaps just 5%) of the estimated 1.5 billion specimens in these collections has any digital record (Cocks et al. 2020). Within European collections, this gap is being addressed by a consortium of natural science collections, who are coordinating in their efforts to make these collections digitally accessible and provide a common digital gateway to facilitate collections access. This network, the Distributed System of Scientific Collections, or "DiSSCo" for short, is in the process of establishing its service infrastructure as it moves to become a legal entity under the European Union "European Research Infrastructure Consortium (ERIC)", which is a specific legal form that facilitates the establishment and operation of European Research Infrastructures (Addink et al. 2019).

As part of the efforts to formalise DiSSCo, a working group was commissioned by the DiSSCo Interim General Assembly to examine the strategic position of DiSSCo with related research infrastructures. Global data infrastructures tend to have specialised niches representing only a narrow segment of the multidimensional biodiversity informatics space. They differ by data type(s) handled and data life cycle phase(s) supported. They may deal with only one data type (e.g. Fishbase, a global species database on fish) or support one or a few links in the data mobilisation chain (e.g. GBIF that collates, integrates and distributes digital data, but does not digitise analogue data). Specialisation may represent a reasonable division of labour at the macro level and be the only feasible way to advance service generation. However, in many countries, the mosaic pattern has been repeated at the national, regional or continental level, necessitated by funding and/or jurisdictional constraints. When this occurs, there is a risk of duplication or actions becoming siloed, hindering effective development.

The worldwide biodiversity informatics landscape is, therefore, composed of numerous elements, which have invested much effort in connecting to provide a complete service array to end-users, but have not always succeeded in avoiding redundancy. The strategic positioning working group of DiSSCo was tasked with examining potential links with related data infrastructures to prove an evidence base that would refine DiSSCo's niche of operation. To minimise these risks for DiSSCo, the working group developed a methodology to examine the niche of DiSSCo and nine related infrastructures. In doing so, we have not only built a comprehensive picture of these infrastructures' activities, both now and into the future, but also a methodology for examining their interrelationships.

## General description

### Purpose

We sought to characterise the current and planned activities performed by major organisations involved in biodiversity informatics, through a quantitative assessment that described not only the many dimensions of their activities, but also their relative technological maturity (referred to as a ‘maturity index’). This maturity index (Table 1) addresses the fact that these organisations are at different stages in their life cycle and many are yet to realise the full maturity of their ambitions.

This framework aimed to allow the results to be actionable, providing insights on where there is likely to be the greatest future contact between the shared ambitions and deepest gaps across the overall landscape of biodiversity informatics activities. At every stage in the data collection process, rigorous efforts were made to standardise the data such that it can be directly compared across each organisation. However, despite the granularity of the framework used to gather data, there may be considerable differences in the activity of organisations operating in precisely the same niche. In such instances, this 'Contact' between the activities of different organisations should signal the need for further investigation, rather than an immediate inference of duplication.

## Project description

### Title

Distributed System of Scientific Collections (DiSSCo) Interim General Assembly: Research Infrastructure Contact Zones Task Force

### Personnel

Wouter Addink, Christos Arvanitidis, Olaf Bánki, Ana Casino, François Dusoulier, Lisa French, Falko Glöckler, Donald Hobern, Aino Juslén, Martin Kalfatovic, Dimitrios Koureas, Patricia Mergen, Joe Miller, Leif Schulman, Vincent Smith, Sarah Vincent, Matt Woodburn

### Design description

A task force was commissioned by the DiSSCo Interim General Assembly to examine the activities and alignment of DiSSCo in relation to the fragmented and complex landscape of related biodiversity informatics organisations and infrastructures. A new framework was developed to survey infrastructures across five categories (data, standards, software, hardware and policy), for nine types of data (specimens, collection descriptions, opportunistic observations, systematic observations, taxonomies, traits, geological data, molecular data and literature) and for seven phases of activity (creation, aggregation, access, annotation, interlinkage, analysis and synthesis). This work was inspired by an early model recently published by the Finnish Biodiversity Information Facility, which depicts biodiversity informatics organisations by the data type supported and the data life cycle phases covered (Schulman et al. 2021).

### Funding

This work was partially supported by the Horizon 2020 Framework Programme of the European Union: H2020-INFRADEV-2019-2020 – DiSSCo Prepare – Grant Agreement No. 871043.

## Sampling methods

### Study extent

A subset of large infrastructures active in biodiversity informatics across Europe and willing to take part, became the focus of our research. These are arguably those infrastructures operating conceptually and geographically closest to the domain of DiSSCo. Nevertheless, the selection excludes many other potentially relevant groups and, in the absence of a global infrastructure registry, it is impossible to fully know how many infrastructures might be missing from this survey. The ten organisations that agreed to participate were: the Distributed System of Scientific Collections (DiSSCo, https://www.dissco.eu/), the Global Biodiversity Information Facility (GBIF, https://www.gbif.org/), the International Barcode of Life (iBOL, https://ibol.org/), the Catalogue of Life (CoL, https://www.catalogueoflife.org/), iNaturalist (https://www.inaturalist.org/), the Biodiversity Heritage Library (BHL, https://www.biodiversitylibrary.org/), the Geoscience Collections Access Service (GeoCASe, https://geocase.eu/), LifeWatch (https://www.lifewatch.eu/), the integrated European Long-Term Ecosystem, critical zone and socio-ecological Research Infrastructure (eLTER, https://elter-ri.eu/) and ELIXIR (https://elixir-europe.org/). By necessity, many of these organisations have global reach, but we particularly focused on related European Strategy Forum on Research Infrastructures (ESFRI) organisations given their proximity in activities, governance model and funding to DiSSCo. Additional organisations within and beyond Europe became interested in the research during the course of our data collection, but we agreed to constrain our initial research to limit the frequency of changes to the survey structure.

### Sampling description

Each infrastructure organisation was sent a personalised data collection template, alongside an extensive Frequently Asked Questions document that outlined the rationale for the work and the methodology. Each organisation was asked to evidence their results and did not have sight of other infrastructures' scores, corresponding to the relative maturity of their activities, during the data collection phase. Considerable efforts were made to allay any concerns about the nature of the survey to minimise the risk of organisations over- or underestimating their scores or declaring a pattern of activity that exceeds their stated actions or ambition. An extensive glossary of terms (Table 1), tightly and clearly defining all the parameters being scored, was included in the data collection template to ensure that there was a common understanding of activities across each organisation and, thus, reinforce the standardisation and comparability of the datasets.

A personalised dataset for each infrastructure was initially populated with preliminary data, based on the Task Force’s initial understanding of each infrastructure's activities. This helped to frame expectations and minimise the data collection burden on the part of the individual(s) completing the survey. In some cases, this preliminary dataset proved a close match to the final verified data submitted and, in a few cases, significantly over- or underestimated the scope and maturity of activities. Regardless, all data providers significantly evidenced their submissions, providing confidence that the data received are a fair and close match to current or planned activities. In several cases, this was further clarified through follow-up discussions with the data provider.

Every effort was made to standardise the interpretation of the terms being assessed. However, some may still be subject to differences in understanding, leading to minor discrepancies in how certain activities were scored. For example, some infrastructures considered their use and development of High-Performance Computing infrastructure, in the context of the survey questions covering hardware development, while others excluded this from their hardware considerations.

### Step description

**Figure 1.**
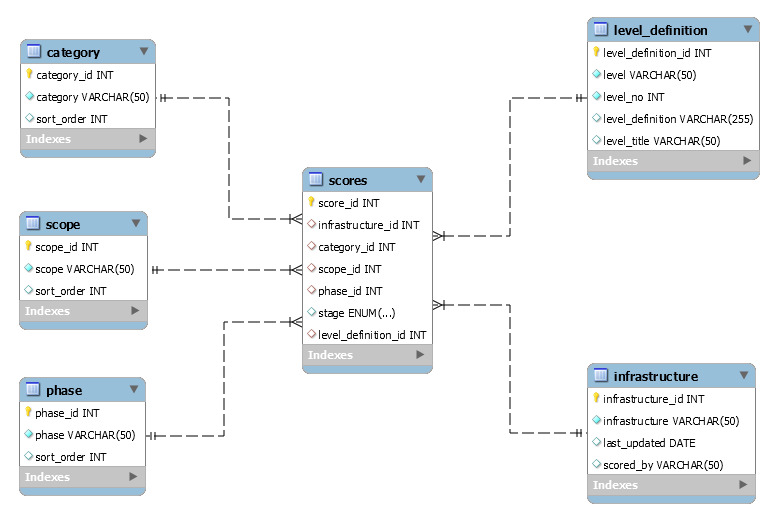
Structure of the Research Infrastructure Contact Zones Database.

A contact zones database was developed to store the survey responses. This was subsequently used to support the data visualisations and analysis of the results. The overall database schema can be found in Fig. 1.

## Geographic coverage

### Description

The research infrastructures described by this dataset have a mix of global and European coverage.

## Usage licence

### Usage licence

Creative Commons Public Domain Waiver (CC-Zero)

## Data resources

### Data package title

Research Infrastructure Contact Zones

### Resource link

https://doi.org/10.5281/zenodo.6138822

### Number of data sets

7

### Data set 1.

#### Data set name

flattened_research_infrastructure_contact_zones.tsv

#### Data format

TSV

#### Description

TSV containing the denormalised, flattened view of the Contact Zones database. Definitions of the terms found in the category, scope and phase columns are found in Table 1.

**Data set 1.** 

Column label	Column description
infrastructure	Name of infrastructure.
category	Name of category.
scope	Name of scope.
phase	Name of phase.
level_current	Maturity index score: current level.
level_ambition	Maturity index score: ambition level.

### Data set 2.

#### Data set name

tbl_scores.tsv

#### Data format

TSV

#### Description

TSV exports of the normalised database tables shown in Fig. 1. These tables include linking fields and other utilitarian/structural elements of the data not included in the flattened version of the data.

**Data set 2.** 

Column label	Column description
score_id	Score ID.
infrastructure_id	Infrastructure ID.
category_id	Category ID.
scope_id	Scope ID.
phase_id	Phase ID.
stage	Indicates if the maturity index score relates to current level or long-term ambition.
level_definition_id	Maturity index level definition ID

### Data set 3.

#### Data set name

tbl_infrastructure.tsv

**Data set 3.** 

Column label	Column description
infrastructure_id	Infrastructure ID.
infrastructure	Name of infrastructure.
last_updated	Date the survey was completed or updated.
scored_by	Name of individual who completed the survey.

### Data set 4.

#### Data set name

tbl_category.tsv

**Data set 4.** 

Column label	Column description
category_id	Category ID.
category	Name of category.
sort_order	Default sort order for category.

### Data set 5.

#### Data set name

tbl_scope.tsv

**Data set 5.** 

Column label	Column description
scope_id	Scope ID.
scope	Name of scope.
sort_order	Default sort order for scope.

### Data set 6.

#### Data set name

tbl_phase.tsv

**Data set 6.** 

Column label	Column description
phase_id	Phase ID.
phase	Name of phase.
sort_order	Default sort order for phase.

### Data set 7.

#### Data set name

tbl_level_definition.tsv

**Data set 7.** 

Column label	Column description
level_definition_id	Maturity index level definition ID.
level	Name of maturity index level.
level_no	Maturity index level number.
level_definition	Definition of maturity index level.
level_title	Full maturity index level title, including name and number.

## Additional information

### Infrastructure Summaries: current and future scope

**Figure 2.**
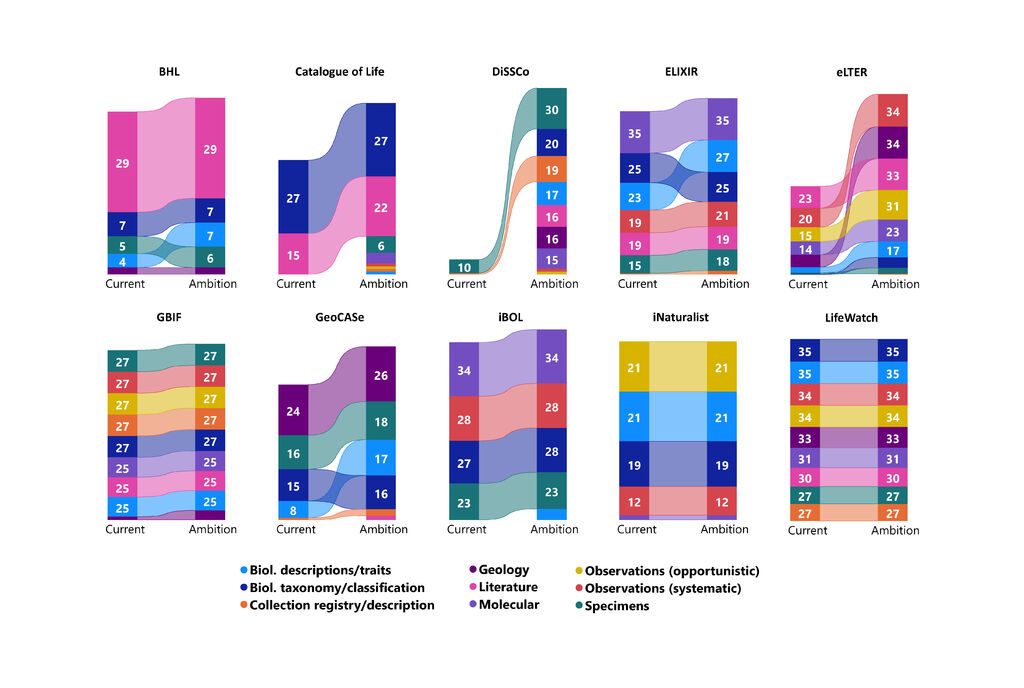
Activity counts with a Maturity Index of 2 and above for each infrastructure within each scope.

The activity levels when viewed by scope shows the subject matter and area of interest of the infrastructures. Table 2 shows the current levels of activity by the scope for each infrastructure, as well as their future ambition. This Table includes a ranking of scope by infrastructure. The changes between current and future (ambition) levels is visualised in Fig. 2.

**Biodiversity Heritage Library**

The Biodiversity Heritage Library is a worldwide consortium and aims to make biodiversity literature openly available through digitisation. This is reflected in their scoring in the contact zones analysis (Fig. 2). Most of their current activities at a Maturity Index of 2 and above (P2 and above) are within the Literature scope (62%, 29 activities) and this remains the focus of BHL's future ambitions.

**Catalogue of Life**

The mission of the Catalogue of Life is to provide a freely accessible list of species across all taxonomic groups. It currently has a tight remit, with P2 and above activities within two scopes: Biological taxonomy/classification (64%, 27 activities) and Literature (36%, 15 activities) (Fig. 2). Catalogue of Life has ambitions to slightly broaden this scope, with some presence in all scope areas apart from Geology and aims to increase its activity within Literature (from 15 activities to 22).

**DiSSCo**

DiSSCo is a new European research infrastructure for natural science collections, aiming to digitally unify European natural science assets (e.g. specimen collections) through their digitisation. It is currently within its preparatory phase, via the DiSSCo Prepare project and this is reflected in the low number of activities currently rated at P2 and above (11 activities), with most of these in the Specimens scope (91%, 10 activities) (Fig. 2). In future, DiSSCo aims to dramatically increase its P2 and above activities from 11 to 137, including the scope of its activities on Specimens (22%, 30 activities), Biological Taxonomy/Classification (15%, 20 activities) and Collection Registry/Description (14%, 19 activities).

**ELIXIR**

ELIXIR aims to coordinate and develop life science resources in Europe, with a particular focus on molecular/genomic bioinformatics resources. It has P2 and above activities in most of the scope areas, including Molecular (26%, 35 activities), Biological Taxonomy/Classification (18%, 25 activities) and Biological Descriptions/Traits (17%, 23 activities) (Fig. 2). ELIXIR aims to keep this broad remit in future, with only a slight increase in the activities in which it has at least a presence - from 135 to 148.

**eLTER**

eLTER is a new European research infrastructure in its preparatory phase of development. It aims to improve the scientific understanding of terrestrial, freshwater and transitional water ecosystems through a socio-ecological approach to studying these systems. eLTER's current activities are highest in Literature (25%, 23 activities) and Observations (Systematic) (22%, 20 activities) (Fig. 2). In the future, Observations (Systematic) remains important (18, 34 activities), but there is an increasing focus on Geology (from 13 to 34 activities). eLTER will continue to have many P2 and above activities within the context of Literature (17%, 33 activities) and will also increase its activities within the Observations (Opportunistic) scope (31 activities, 16%).

**GBIF**

GBIF is a global network and data infrastructure that provides open access to data about life on Earth, as well as common standards and open-source tools to enable the sharing of information about where species have been recorded. It currently has a large number of activities at P2 and above (214 out of 315 possible activities) (Fig. 2). GBIF has the least concentration of activities within the scope of Geology. GBIF plans to continue this spread of activities in future and aims to increase its presence in Geology (from 4 to 12 activities).

**GeoCASE**

GeoCASE is designed to make data on collections of minerals, rocks, meteorites and fossils easily accessible online. In this regard, GeoCASE aims to be the Earth Science counterpart to GBIF. This mission is reflected in the P2 and above activities that GeoCASe have recorded in this dataset, with most of its ambition scores within the scope of Geology (28%, 24 activities), Specimens (25%, 16 activities) and Biological Taxonomy/Classification (23%, 15 activities) (Fig. 2). GeoCASE plans to maintain its presence in these areas, as well as increase activity within Biological Description/Traits (from 8 to 16 activities).

**iBOL**

iBOL is a global research alliance that builds DNA barcode reference libraries, sequencing facilities and informatics platforms with the aim to discover and identify multicellular life. iBOL’s current P2 and above activities are within four scope areas: Molecular (30%, 34 activities), Observations (systematic) (25%, 28 activities), Biological Taxonomy/Classification (24%, 27 activities) and Specimens (21%, 23 activities) (Fig. 2). This continues in future, with not much change in the activities it aims to be at P2 and above, although it does aim to slightly expand into the Biological Description/Traits scope (7 activities).

**iNaturalist**

iNaturalist allows naturalists and citizen scientists to record their observations of biodiversity via mobile apps or through their website, with their research-grade findings shared with GBIF. The majority of iNaturalist’s P2 and above activities currently focused on Observations (Opportunistic) (28%, 21 activities), Biological Descriptions/Traits (28%, 21 activities) and Biological Taxonomy/Classification (25%, 19 activities) (Fig. 2). iNaturalist is a well-established infrastructure with a relatively narrow and distinct niche and does not aim to widen the breadth of its P2 and above activities in future.

**LifeWatch**

LifeWatch is a European Research Infrastructure Consortium (ERIC) that provides e-services to biodiversity and ecosystem researchers, helping to address planetary challenges. LifeWatch currently has a P2 and above in most activities relevant to the biodiversity informatics domain, with a presence in most activities in every scope (286 out of 315 activities). Although LifeWatch does not plan to significantly increase this breadth in the future (Fig. 2), this survey was completed before the development of a new five-year Strategic Working Plan, launched in June 2022.

### Measuring ambition: how the development of these infrastructures will change the biodiversity informatics landscape

**Figure 3.**
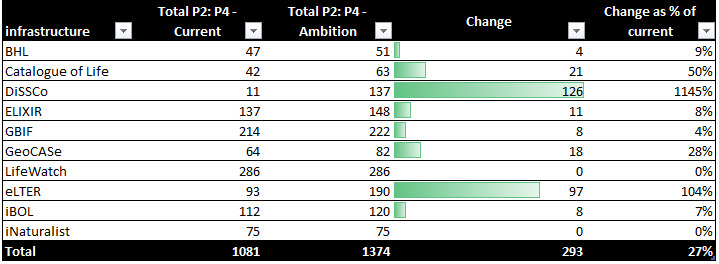
Change in Total Activities (Maturity Index 2 and above) by Infrastructure.

Fig. 3 shows the current activity levels and the future ambitions of each infrastructure, with a count of the number of activities each infrastructure has at Maturity Index Level of P2 (presence) and above. GBIF and LifeWatch have the highest number of activities and could be considered generalists, with both showing a presence in over 200 current activities. iBOL and ELIXIR both have a presence of over 100 activities, with a degree of specialisation, with DiSSCo and eLTER planning to fall within a similar range of activities as they deliver on their development roadmaps. BHL, CoL, GeoCASe and iNaturalist are much more specialised, each operating with a narrow focus of activities (all below 85 activities).

DiSSCo and eLTER are both new infrastructures in the early stages of development and show the greatest difference in activity between current levels and future ambition. Two of the more specialist infrastructures, GeoCASe and Catalogue of Life, also have proportionally ambitious plans to expand their activities compared to other infrastructures. The introduction of two new infrastructures, which aim to actively expand their activity levels, will likely result in a changing dynamic from the current landscape and require new collaborations and coordination. Consideration of existing mechanisms of collaboration with specialist organisations like GeoCASe and Catalogue of Life will also be beneficial as they start to broaden their activities.

**Figure 4.**
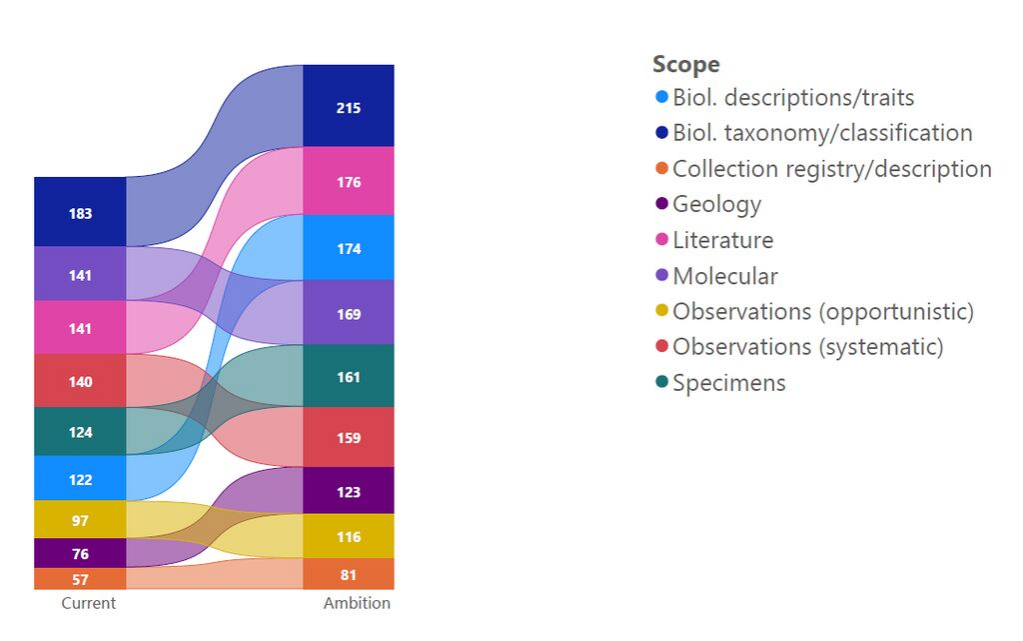
Change in the total activities (Maturity Index P2 and above) across all infrastructures from current to ambition.

An analysis of the current and future activities within each scope shows the changing nature of the research infrastructure landscape (Fig. 4). Observations (both opportunistic and systematic) and Geology are the scopes with the lowest current activity levels and this is likely to remain the case in future. Biological Taxonomy/Classification has the highest activity levels, both now and in future. The highest increase in activity can be found in Biological Description/Traits and there may be a need to strengthen collaboration between infrastructures in this space going forward.

**Figure 5.**
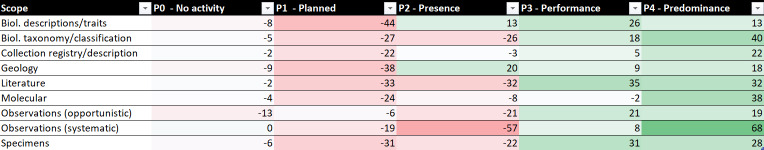
Change in the count of activities within each scope rated at Maturity Index Level between current and ambition.

It is also possible to look at how the landscape plans to shift in future and whether there is an overall increase in maturity levels in the activities for each scope. All scopes show an increase in the number of activities that will be at the P3 'Performance' and P4 'Predominance' levels in future (Fig. 5). Within the Geology and Biological Descriptions/Traits scope, there is also an increase in activities rated at 'P2 - Presence'. This is due to these two scopes being relatively immature in comparison to other scope areas, with more potential activities that are currently absent (P0).

**Figure 6.**
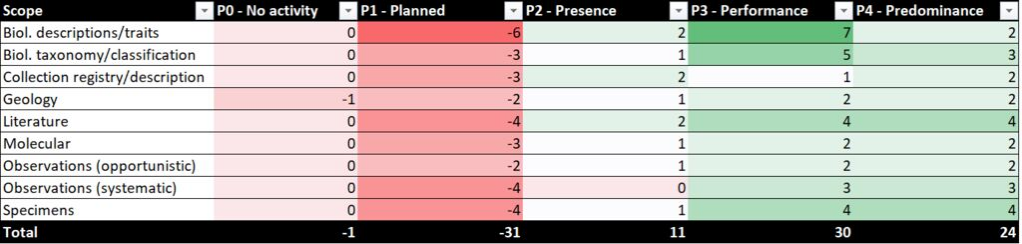
Change in the count of infrastructures within each scope by Maturity Index Level between current and ambition.

There is a notable shift in Observations (Systematic) with activities moving from P2 'Presence' to P4 'Predominance' and the Molecular scope also showing a large movement towards activities rated P4 'Predominance'. This change is primarily through an increase in activity by a small number of infrastructures (Fig. 6) and collaboration in this space between these infrastructures would likely be beneficial. A large number of infrastructures have moved to P3 'Performance' within Biological Descriptions/Traits and, as mentioned above, there is a high increase in activity within this scope. This is likely to be an area where collaboration and cooperation between the majority of infrastructures will be required to ensure alignment and synergy in activity.

**Future perspectives**

This dataset and methodology for quantitatively assessing present and planned infrastructure activities hold considerable promise to support cooperation and planning amongst biodiversity informatics research infrastructures. In the first instance, expanding the dataset by adding closely related infrastructures and networks, such as TDWG (Biodiversity Information Standards; https://www.tdwg.org/), MIRRI (Microbial Resource Research Infrastructure; https://www.mirri.org/), iDigBio (Integrated Digitized Biocollections; https://www.idigbio.org/) and ALA (Atlas of Living Australia; https://www.ala.org.au/), would be beneficial to provide a more complete picture of the biodiversity informatics landscape. A more complete assessment of eligible infrastructures might draw on recent reviews of the biodiversity informatics domain, starting with the requirements set out in the OECD Megascience report from 1999 on biological informatics (OECD 1999).

Another limitation of our approach is that the methodology is dependent on self-assessments that were only validated and reviewed by the survey team. This could be improved through a larger community survey by asking more independent network stakeholders to assess a research infrastructure's coverage and maturity and by comparing these results with the self-reported scores of the research infrastructure. In the longer term, more automated methodologies, such as those used by various FAIR-metrics working groups within the Research Data Alliance (https://www.rd-alliance.org/), European Open Science Cloud (https://eosc.eu/) and Go-FAIR (https://www.go-fair.org/) communities, might provide inspiration for building more objective evaluation criteria.

More refined data visualisations, dynamically constructed off of a growing dataset of research infrastructures would also be useful to support the strategic development of service provision by these infrastructures, as well as identifying gaps in the landscape. Further generalisation of the method, including an expansion of the Scope, Phase and Category terms to encompass activities in other domains beyond biodiversity informatics, has the potential to broaden the application of this approach, potentially providing an evidence base when considering strategic investments in a much wider range of research infrastructures. For example, this approach has the potential to support investment decisions by funders (e.g. ESFRI, the European Strategy Forum on Research Infrastructures; https://www.esfri.eu/), which is a strategic instrument used in Europe to develop the scientific integration of research infrastructures. The dataset and tool also have potential for associated infrastructures like EOSC, the European Open Science Cloud (https://eosc-portal.eu/). EOSC's efforts to address the cloud-computing need of other infrastructures, may benefit from a deeper understanding of the current and future of potential user communities when planning targetting application of their services.

## Figures and Tables

**Table 1.** Definitions of terms provided to the biodiversity data infrastructures and used during the assessment process.

**Category**	**Term**	**Definition**
General	Organisation	An entity – such as a company, an institution or an association – comprising one or more people and having a particular purpose. In the context of this framework, this is the entity whose activity is being scored.
	Type	A high-level class of information associated with a physical specimen held within a natural science collection.
	Phase	A stage with the data processing lifecycle.
	Infrastructure	The set of fundamental content, facilities, systems or services necessary for a community to function.
	Maturity Index	A measurement system used to assess the maturity level of a particular activity, domain or technology.
	Evidence	Examples relevant to the major 'Type' (not 'Phase') of activity, given as short unstructured text remarks and/or web links to further information.
Scope	Specimens	An evidential record of an individual, item or part of a natural science collection.
	Collection registry/description	Metadata used to describe any set of individuals, items or parts (specimens) that form a whole or part of a natural science collection.
	Observations (opportunistic)	An evidential record of an unplanned encounter with an individual organism at a particular time and place.
	Observations (systematic)	An evidential record of an encounter with an individual organism at a particular time and place as part of a programme of study.
	Biological taxonomy/classification	Any activities associated with the branch of science that encompasses the description, identification, nomenclature and classification of organisms.
	Biological descriptions/traits	The non-molecular phenotype of a biological entity, in the form of a text description, statement, multimedia or dataset.
	Geology	Any aspect of the characterisation (including Earth or planetary system science) of rocks and minerals of any origin, in the form of a text description, statement or dataset.
	Molecular	Any aspect of the structure, function, evolution, mapping and editing of an organism's DNA or RNA nucleotides.
	Literature	Any non-fiction scholarly writing or metadata associated with such writing, concerning any aspect of the natural world.
Phase	Create	The first stage in the data life cycle in which an initial digital representation is created.
	Aggregate	The bringing together of a group, body or mass composed of many distinct parts or individuals.
	Access	The "ability to access" and benefit from some system or Accept entity.
	Annotate	The addition of extra information associated with a particular point in any data, information or knowledge.
	Interlink	The connection of things (e.g. entities in a database).
	Analyse	To subject to scientific analysis.
	Synthesis	The combining of often diverse conceptions into a coherent whole to create new knowledge.
Category	Data/Content	Factual information used as a basis for reasoning, discussion or calculation.
	Standards	The rules (format and meaning) by which data are described, recorded and exchanged.
	Software	Any set of programmes, procedures and routines associated with the operation of a computer system.
	Hardware	Tools, machinery and other durable equipment (e.g. computers and storage) associated with any phase of activity.
	Policy/Culture	The community networks and agreed practices to make our activities an openly shared, freely available, connected resource.
Maturity Index	P0 - No activity/inapplicable	No current/planned activity or inapplicable to an organisation's operations.
	P1 - Planned	Named a strategy, roadmap or outline development as a proof of concept (evidenced through documentation or a prototype solution).
	P2 - Presence	Addresses part of the domain/problem set served, sometimes as a dependency to addressing other issues and in use (evidenced through the use of the solution beyond the developing organisation).
	P3 - Performance	Addresses a majority/full scope of the domain it serves and in widespread use (evidenced through the richness of feature set and widespread use).
	P4 - Predominance	A domain leader to which all other innovators would aspire to or work with, addressing the full scope of the domain and sustained through continuous improvement (evidenced through market share).

**Table 2.** Infrastructure priorities: scope-focus per infrastructure, ranked by proportion of activities at level P2-P4.

**Infrastructure**	**Rank - Current**	**Top Scopes - Current**	**Activities P2-P4 - Current**	**Scope % - Current**	**Rank - Ambition**	**Top Scopes - Ambition**	**Activities P2-P4 - Ambition**	**Scope % - Ambition**
BHL	1	Literature	29	62%	1	Literature	29	57%
BHL	2	Biol. taxonomy/classification	7	15%	2	Biol. descriptions/traits	7	14%
BHL	3	Specimens	5	11%	2	Biol. taxonomy/classification	7	14%
BHL	4	Biol. descriptions/traits	4	9%	3	Specimens	6	12%
BHL	5	Geology	2	4%	4	Geology	2	4%
BHL		Observations (systematic)	0	0%		Collection registry/description	0	0%
BHL		Molecular	0	0%		Observations (opportunistic)	0	0%
BHL		Collection registry/description	0	0%		Observations (systematic)	0	0%
BHL		Observations (opportunistic)	0	0%		Molecular	0	0%
Catalogue of Life	1	Biol. taxonomy/classification	27	64%	1	Biol. taxonomy/classification	27	43%
Catalogue of Life	2	Literature	15	36%	2	Literature	22	35%
Catalogue of Life		Observations (opportunistic)	0	0%	3	Specimens	6	10%
Catalogue of Life		Observations (systematic)	0	0%	4	Molecular	4	6%
Catalogue of Life		Specimens	0	0%	5	Collection registry/description	1	2%
Catalogue of Life		Molecular	0	0%	5	Biol. descriptions/traits	1	2%
Catalogue of Life		Collection registry/description	0	0%	5	Observations (opportunistic)	1	2%
Catalogue of Life		Geology	0	0%	5	Observations (systematic)	1	2%
Catalogue of Life		Biol. descriptions/traits	0	0%		Geology	0	0%
DiSSCo	1	Specimens	10	91%	1	Specimens	30	22%
DiSSCo	2	Collection registry/description	1	9%	2	Biol. taxonomy/classification	20	15%
DiSSCo		Observations (opportunistic)	0	0%	3	Collection registry/description	19	14%
DiSSCo		Observations (systematic)	0	0%	4	Biol. descriptions/traits	17	12%
DiSSCo		Biol. descriptions/traits	0	0%	5	Geology	16	12%
DiSSCo		Molecular	0	0%	5	Literature	16	12%
DiSSCo		Biol. taxonomy/classification	0	0%	6	Molecular	15	11%
DiSSCo		Geology	0	0%	7	Observations (systematic)	2	2%
DiSSCo		Literature	0	0%	8	Observations (opportunistic)	2	2%
ELIXIR	1	Molecular	35	26%	1	Molecular	35	24%
ELIXIR	2	Biol. taxonomy/classification	25	18%	2	Biol. descriptions/traits	27	18%
ELIXIR	3	Biol. descriptions/traits	23	17%	3	Biol. taxonomy/classification	25	17%
ELIXIR	4	Observations (systematic)	19	14%	4	Observations (systematic)	21	14%
ELIXIR	4	Literature	19	14%	5	Literature	19	13%
ELIXIR	5	Specimens	15	11%	6	Specimens	18	12%
ELIXIR	6	Collection registry/description	1	1%	7	Collection registry/description	3	2%
ELIXIR		Geology	0	0%		Observations (opportunistic)	0	0%
ELIXIR		Observations (opportunistic)	0	0%		Geology	0	0%
eLTER	1	Literature	23	25%	1	Observations (systematic)	34	18%
eLTER	2	Observations (systematic)	20	22%	1	Geology	34	18%
eLTER	3	Observations (opportunistic)	15	16%	2	Literature	33	17%
eLTER	4	Molecular	14	15%	3	Observations (opportunistic)	31	16%
eLTER	5	Geology	13	14%	4	Molecular	23	12%
eLTER	6	Biol. descriptions/traits	6	7%	5	Biol. descriptions/traits	17	9%
eLTER	7	Biol. taxonomy/classification	1	1%	6	Biol. taxonomy/classification	11	6%
eLTER	7	Specimens	1	1%	7	Specimens	6	3%
eLTER		Collection registry/description	0	0%	8	Collection registry/description	1	1%
GBIF	1	Observations (systematic)	27	13%	1	Specimens	27	12%
GBIF	1	Observations (opportunistic)	27	13%	1	Biol. taxonomy/classification	27	12%
GBIF	1	Collection registry/description	27	13%	1	Collection registry/description	27	12%
GBIF	1	Specimens	27	13%	1	Observations (systematic)	27	12%
GBIF	1	Biol. taxonomy/classification	27	13%	1	Observations (opportunistic)	27	12%
GBIF	2	Biol. descriptions/traits	25	12%	2	Molecular	25	11%
GBIF	2	Literature	25	12%	2	Literature	25	11%
GBIF	2	Molecular	25	12%	2	Biol. descriptions/traits	25	11%
GBIF	3	Geology	4	2%	3	Geology	12	5%
GeoCASe	1	Geology	24	38%	1	Geology	26	32%
GeoCASe	2	Specimens	16	25%	2	Specimens	18	22%
GeoCASe	3	Biol. taxonomy/classification	15	23%	3	Biol. descriptions/traits	17	21%
GeoCASe	4	Biol. descriptions/traits	8	13%	4	Biol. taxonomy/classification	16	20%
GeoCASe	5	Collection registry/description	1	2%	5	Collection registry/description	3	4%
GeoCASe		Observations (systematic)	0	0%	6	Literature	2	2%
GeoCASe		Literature	0	0%		Observations (opportunistic)	0	0%
GeoCASe		Molecular	0	0%		Molecular	0	0%
GeoCASe		Observations (opportunistic)	0	0%		Observations (systematic)	0	0%
iBOL	1	Molecular	34	30%	1	Molecular	34	28%
iBOL	2	Observations (systematic)	28	25%	2	Biol. taxonomy/classification	28	23%
iBOL	3	Biol. taxonomy/classification	27	24%	2	Observations (systematic)	28	23%
iBOL	4	Specimens	23	21%	3	Specimens	23	19%
iBOL		Biol. descriptions/traits	0	0%	4	Biol. descriptions/traits	7	6%
iBOL		Observations (opportunistic)	0	0%		Geology	0	0%
iBOL		Collection registry/description	0	0%		Literature	0	0%
iBOL		Geology	0	0%		Observations (opportunistic)	0	0%
iBOL		Literature	0	0%		Collection registry/description	0	0%
iNaturalist	1	Observations (opportunistic)	21	28%	1	Biol. descriptions/traits	21	28%
iNaturalist	1	Biol. descriptions/traits	21	28%	2	Observations (opportunistic)	21	28%
iNaturalist	2	Biol. taxonomy/classification	19	25%	3	Biol. taxonomy/classification	19	25%
iNaturalist	3	Observations (systematic)	12	16%	4	Observations (systematic)	12	16%
iNaturalist	4	Molecular	2	3%	5	Molecular	2	3%
iNaturalist		Specimens	0	0%		Geology	0	0%
iNaturalist		Collection registry/description	0	0%		Literature	0	0%
iNaturalist		Geology	0	0%		Specimens	0	0%
iNaturalist		Literature	0	0%		Collection registry/description	0	0%
LifeWatch	1	Biol. taxonomy/classification	35	12%	1	Biol. descriptions/traits	35	12%
LifeWatch	1	Biol. descriptions/traits	35	12%	1	Biol. taxonomy/classification	35	12%
LifeWatch	2	Observations (opportunistic)	34	12%	2	Observations (systematic)	34	12%
LifeWatch	2	Observations (systematic)	34	12%	2	Observations (opportunistic)	34	12%
LifeWatch	3	Geology	33	12%	3	Geology	33	12%
LifeWatch	4	Molecular	31	11%	4	Molecular	31	11%
LifeWatch	5	Literature	30	11%	5	Literature	30	11%
LifeWatch	6	Specimens	27	9%	6	Collection registry/description	27	9%
LifeWatch	6	Collection registry/description	27	9%	6	Specimens	27	9%
